# Supplementary material for: Point-of-care prognostication in moderate Covid-19: Analytical validation and prognostic accuracy of a soluble urokinase plasminogen activator receptor (suPAR) rapid test
Source: PLOS Glob Public Health. 2023 Aug 21;3(8):e0001538. doi: 10.1371/journal.pgph.0001538 (PMC10441780; doi:10.1371/journal.pgph.0001538)
Supplement: Attachment — Submitted filename: PRIORITISE_PGPH_suPAR_RDT_rebuttal.docx [file pgph.0001538.s002.docx]

**4 January 2023**

Dear PLoS Global Public Health editorial team,

**Re: PGPH-D-22-01887 – Point-of-care prognostication in moderate Covid-19: analytical validation and prognostic accuracy of a soluble urokinase plasminogen activator receptor (suPAR) rapid test**

Thank you for considering our article for publication in the *Diagnostics in Global Heath* collection in PLoS Global Public Health. We appreciate the time your team have taken to consider our submission. Naturally, we are disappointed with the outcome. However, we do have concerns with regards the reasons cited for declining our submission, which suggest there may have been a significant misunderstanding of our work.

We note that the editorial board members were concerned that our abstract was not clear and specifically that; (i) we had not described the aims or hypothesis of our study; (ii) that no control group was included; and (iii) that no gold standard was mentioned. However, we do state our aim (lines 45/46: “*evaluate the analytical performance and diagnostic accuracy of a commercially-available rapid diagnostic test for suPAR*”) and the gold standard is reported (lines 46/47: “*using an ELISA as the reference method*”). Furthermore, as this is a prognostic study, the control group is by definition made up of the patients who did not progress to meet the primary outcome, of which there are 333 patients. It is important to note that there would not be a control group of ‘disease-free’ individuals in the same way that one might expect if we were conducting a diagnostic study, for example, evaluating a rapid diagnostic test for the confirmation of a particular pathogen (e.g. dengue NS1 rapid test or malaria RDT).

We sincerely apologise if our abstract did not make some aspects of our study clear. We acknowledge this feedback from the editorial team and enclose a tracked changes version of our manuscript. In particular, we have made our aim more explicitly clear and we have included our hypothesis in the abstract (previously this was only stated at the end of the Introduction). We have also changed the words “*diagnostic accuracy*” to “*prognostic accuracy*” throughout the manuscript to avoid any confusion as to the type of study that we conducted. We had originally used the term “diagnostic accuracy” as this is used in the STARD guidelines (and informally for all sorts of ‘diagnostic’ test evaluations, whether they be diagnostic or prognostic tests). However, we do appreciate that this may have caused confusion and have therefore now used a more scientifically precise term.

Having read the comments from the editorial board, we do feel there has been a misunderstanding with regards the nature of our study and we would be very grateful if you might reconsider sending our manuscript for peer-review. Thank you for considering our request.

Yours Sincerely,

**Dr. Sakib Burza MB BChir MRCGP MSc PhD**

Medical Advisor, New Delhi, MSF India

Honorary Associate Professor, Department of Clinical Research, London School of Hygiene & Tropical Medicine

**Dr. Arjun Chandna BA MBBS MRCP AFHEA**

Research Physician, Cambodia Oxford Medical Research Unit, Siem Reap, Cambodia

Wellcome Trust Doctoral Fellow, Centre for Tropical Medicine & Global Health, University of Oxford
